# Editorial: Effects of Game and Game-Like Training on Neurocognitive Plasticity

**DOI:** 10.3389/fnhum.2016.00123

**Published:** 2016-03-30

**Authors:** Guido P. H. Band, Chandramallika Basak, Heleen A. Slagter, Michelle W. Voss

**Affiliations:** ^1^Leiden Institute for Brain and CognitionLeiden, Netherlands; ^2^School of Behavioral and Brain Sciences, University of Texas at DallasDallas, TX, USA; ^3^Department of Psychology, University of AmsterdamAmsterdam, Netherlands; ^4^Department of Psychological and Brain Sciences, University of IowaIowa City, IA, USA

**Keywords:** videogame, learning, training effects, cognitive control, prefrontal cortex, neuromodulators, working memory, plasticity

Playing videogames is a widely practiced leisure activity. Because of the time spent playing and the interactive content, game-guided learning could potentially supplement or replace more traditional mediums for learning and rehabilitation. There are many shades of gray between pure cognitive training, where the participant pursues the goal of a cognitive benefit directly, and game-based training, where training is contained in activities with their own intrinsic motivational value. Finding the optimal approach requires a better understanding of the underlying mechanisms (cf. Karbach and Schubert, 2013). We therefore conducted a research topic entitled “Effects of game and game-like training on neurocognitive plasticity,” composed of eight empirical papers, an opinion paper and a review that contribute to the quest for effective ways to employ game-guided learning.

Several studies have shown benefits from videogames for cognitive functions such as visual attention (Green and Bavelier, 2003, 2007), and multitasking (Basak et al., 2008; Anguera et al., 2013). However, transfer of cognitive training to criterion tasks is not always observed. For a reliable picture of game effects, methodological rigor is necessary; to recalibrate positive findings (Redick and Webster) and to give way to publication of reliable null-results (Boot et al., 2011). Optimizing methodology is an ongoing process, as Zelinski et al. demonstrated using structural equation modeling to validly evaluate transfer.

How do games make training more effective? Games can help overcome cognitive limitations by employing adaptive difficulty, informative feedback, and a sufficient dosage (Mishra and Gazzaley). But the positive role that is often attributed to increased motivation (Habgood and Ainsworth, 2011), prolonged immersion and fun inherent in games may be overestimated. Wang et al. tested the optimal way to schedule a working memory training intervention. They found that transfer from training to fluid intelligence scores occurred only after spreading training sessions over 20 days, and not after any of the denser schedules. Thus, long game sessions may not be the most efficient way to learn. Katz et al. performed a systematic study to identify motivational game features that could boost children's working memory. Surprisingly, game elements such as real-time score displays modulated learning only negatively. This illustrates that mere addition of game elements to disguise the burden of active training works counterproductive. Rather, game-guided training should comply with the same evidence-based principles as traditional forms of training (e.g., Schmidt and Bjork, 1992; Pashler et al., 2007).

What are the cognitive mechanisms that make games beneficial for training? Both game designers and trainers acknowledge the difficulty of merging gaming with training features. When there is too much focus on cognitive benefit, game elements may fail to raise motivation. Conversely, when game elements dominate, they exert a cognitive load at the expense of capacity available for target training (cf. Sweller et al., 1998), or at least draw attention away from the target training. It is fair to assume that the amount of learning is a function of the intensity and the focus of attention for relevant learning material. Indeed, Nikolaidis et al. demonstrated that individual differences in the working memory related brain response to game training are predictive of post-intervention changes on the Sternberg memory search task.

The influence of attentional focus and intensity in games can be translated to game effects on neuromodulation (e.g., Deveau et al.). Reward is a common game ingredient, associated with the release of dopamine (Koepp et al., 1998) and consequently, new habit formation. Gaming elements can help preserve reward responsiveness as measured in ventral striatum after training (Lorenz et al.). Furthermore, games often trigger arousal, characterized by phasic norepinephrine release, which facilitates attention and memory encoding (Tully and Bolshakov, 2010). Note, however, that trainees vary in neuromodulatory activity across age groups and between trainees with and without psychopathology (e.g., schizophrenia, ADHD). Thus, game mechanics should ideally adapt to individual differences in optimal levels of reward, reward responsiveness (cf. Gray, 1982) and arousal.

A different form of brain stimulation targets brain activity at EEG spectral bands associated with attention, working memory and control. Reedijk et al. used binaural beats to entrain target frequencies alpha (10 Hz) or gamma (40 Hz), which led to improved divergent thinking scores, at least for participants with lower dopamine levels. The same reasoning, that entrainment of the brain activity spectrum can improve cognitive performance, is followed in applying neurofeedback and transcranial AC stimulation. Mishra and Gazzaley argued that integrating such entrainment techniques into games, the closed-loop approach, may be able to boost game efficacy.

Finally, games can target qualitative changes in task performance. Bavelier et al. (2012) argued that transfer occurs if games help in “learning to learn,” which involves better selection of relevant information and recognition of a problem structure. Stamenova et al. found data challenging this principle, however. They trained older adults to better distinguish targets from foils in a recollection training task. Although false alarm rates decreased, there was no transfer to non-trained memory tests, possibly because the stimulus set used for training was too limited. Generalization of learning increases as variability in input and training tasks increases (Schmidt and Bjork, 1992). High variability at the level of the learning material likely fosters learning at a more abstract level of representation (Green and Bavelier, 2008; Slagter, 2012). Another example of a qualitative cognitive change by game-guided training is the observation of Connors et al. who trained blind participants navigation through a building, using either audio- or game-guided training. Although standard performance improved equally for both groups, the game-guided training group could apply the same spatial knowledge faster and more flexibly, suggesting deeper or more implicit learning.

In sum, game elements have clear potential to improve training via multiple mechanisms. Perhaps the largest challenge in optimizing the efficacy of game-guided training is to gear interventions to individual differences. Variable brain anatomy, connectivity, baseline performance, age, neuromodulation, and strategy use can all modulate effects of game training parameters. As more is known about the neurocognitive properties of the trainee, games can be adapted accordingly. Ultimately, this approach would merge game design with insights from cognitive neuroscience and educational neuroscience.

## Author contributions

All authors listed, have made substantial, direct and intellectual contribution to the work, and approved it for publication.

## Funding

The third author was supported by a VIDI award of the Netherlands Organisation for Scientific Research.

### Conflict of interest statement

The authors declare that the research was conducted in the absence of any commercial or financial relationships that could be construed as a potential conflict of interest.

## References

[B1] Anguera J. A. Boccanfuso J. Rintoul J. L. Al-Hashimi O. Faraji F. Janowich J.. (2013). Video game training enhances cognitive control in older adults. Nature 501, 97–101. 10.1038/nature12486 24005416 PMC3983066

[B2] Basak C. Boot W. R. Voss M. W. Kramer A. F. (2008). Can training in a real-time strategy videogame attenuate cognitive decline in older adults? Psychol. Aging 23, 765–777. 10.1037/a0013494 19140648 PMC4041116

[B3] Bavelier D. Green C. S. Pouget A. Schrater P. (2012). Brain plasticity through the life span: learning to learn and action video games. Ann. Rev. Neurosci. 35, 391–416. 10.1146/annurev-neuro-060909-152832 22715883

[B4] Boot W. R. Blakely D. P. Simons D. J. (2011). Do action video games improve perception and cognition? Front. Psychol. 2:226. 10.3389/fpsyg.2011.00226 21949513 PMC3171788

[B5] Gray J. A. (1982). The Neuropsychology of Anxiety: An Enquiry into the Functions of the Septo-Hippocampal System. Oxford: Oxford University Press.

[B6] Green C. S. Bavelier D. (2003). Action video game modifies visual selective attention. Nature 423, 534–537. 10.1038/nature01647 12774121

[B7] Green C. S. Bavelier D. (2007). Action-video-game experience alters the spatial resolution of vision. Psychol. Sci. 18, 88–94. 10.1111/j.1467-9280.2007.01853.x 17362383 PMC2896830

[B8] Green C. S. Bavelier D. (2008). Exercising your brain: a review of human brain plasticity and training- induced learning. Psychol. Aging 23, 692–701. 10.1037/a0014345 19140641 PMC2896818

[B9] Habgood M. P. J. Ainsworth S. E. (2011). Motivating children to learn effectively: exploring the value of intrinsic integration in educational games. J. Learn. Sci. 20, 169–206. 10.1080/10508406.2010.508029

[B10] Karbach J. Schubert T. (2013). Training-induced cognitive and neural plasticity. Front. Hum. Neurosci. 7:48. 10.3389/fnhum.2013.00048 23437015 PMC3579194

[B11] Koepp M. J. Gunn R. N. Lawrence A. D. Cunningham V. J. Dagher A. Jones T.. (1998). Evidence for striatal dopamine release during a video game. Nature 393, 266–268. 10.1038/30498 9607763

[B12] Pashler H. Rohrer D. Cepeda N. J. Carpenter S. K. (2007). Enhancing learning and retarding forgetting: choices and consequences. Psychon. Bull. Rev. 14, 187–193. 10.3758/BF03194050 17694899

[B13] Schmidt R. A. Bjork R. A. (1992). New conceptualizations of practice: common principles in three paradigms suggest new concepts for training. Psychol. Sci. 3, 207–217. 10.1111/j.1467-9280.1992.tb00029.x

[B14] Slagter H. A. (2012). Conventional working memory training may not enhance intelligence. Trends Cogn. Sci. 16, 582–583. 10.1016/j.tics.2012.10.001 23089361

[B15] Sweller J. Van Merrienboer J. J. G. Paas F. G. W. C. (1998). Cognitive architecture and instructional design. Educ. Psychol. Rev. 10, 251–296. 10.1023/A:1022193728205

[B16] Tully K. Bolshakov V. Y. (2010). Emotional enhancement of memory: how epinephrine enables synaptic plasticity. Mol. Brain 3:15. 10.1186/1756-6606-3-15 20465834 PMC2877027

